# Image Quality Assessment Methods in Clinical Diagnostic Imaging: Overview of Current Technical Perspectives

**DOI:** 10.3390/diagnostics16081183

**Published:** 2026-04-16

**Authors:** Rafal Obuchowicz, Adam Piórkowski, Michał Strzelecki

**Affiliations:** 1 Department of Diagnostic Imaging, Jagiellonian University Medical College, 30-663 Krakow, Poland; 2 Department of Biocybernetics and Biomedical Engineering, AGH University of Krakow, 30-059 Krakow, Poland; pioro@agh.edu.pl; 3 Institute of Electronics, Łódź University of Technology, 93-590 Łódź, Poland; michal.strzelecki@p.lodz.pl

## 1. Introduction

Diagnostic imaging is a cornerstone of modern medicine, supporting disease detection, therapy planning, treatment monitoring, and outcome assessment across virtually all clinical specialties. Regardless of the imaging modality employed—radiography, computed tomography (CT), magnetic resonance imaging (MRI), or ultrasonography—the clinical usefulness of an examination ultimately depends on the quality of the acquired images. Image quality determines not only whether pathological changes can be detected but also whether their extent, severity, and clinical relevance can be assessed with sufficient confidence.

Although image quality is often treated as a technical characteristic defined by physical system parameters, from a clinical perspective, image quality is best understood as a task-dependent attribute. The requirements for image quality substantially vary between diagnostic scenarios, such as lesion detection, quantitative tissue characterization, functional assessment, and longitudinal follow-up. Consequently, image quality cannot be adequately described by a single universal metric; instead, image quality reflects a balance among spatial resolution, contrast, noise, and artifact suppression tailored to a specific clinical task. This task-driven view of image quality forms the basis for the medical perspective discussed in Section 2.1 and further outlined in Section 2.2 and Section 2.3.

Assessing image quality has become increasingly complex. Advances in detector technology, reconstruction algorithms, and post-processing methods—particularly those based on artificial intelligence (AI)—have enabled substantial reductions in radiation dose and acquisition times as well as the enhanced visualization of anatomical structures. At the same time, these developments have introduced new challenges, including altered noise characteristics, reconstruction-dependent image appearance, domain shift in multicenter datasets, and the risk of algorithm-induced artifacts. As a result, classical image quality metrics, such as the signal-to-noise ratio (SNR) or contrast-to-noise ratio (CNR), often do not sufficiently capture diagnostic reliability in contemporary imaging workflows. These limitations motivated the technical considerations outlined in Section 2.4 and the discussion of advanced quality assessment approaches in Section 2.5.

The growing integration of machine learning and deep learning into diagnostic imaging further amplifies the importance of robust image quality assessment. Automated algorithms increasingly rely on subtle image features that may be imperceptible to the human observer but are highly sensitive to acquisition protocols, reconstruction settings, and scanner-specific characteristics. In this context, image quality directly affects not only human interpretation but also the performance, generalizability, and safety of AI-based decision support systems. The clinical implications of these developments, including their impact on diagnostic confidence and patient management, are addressed in Section 2.6.

Against this background, this Special Issue, Image Quality Assessment Methods in Radiography, Computed Tomography and Magnetic Resonance, gathers contributions that address image quality from complementary methodological and clinical perspectives. The published studies can be broadly grouped into three thematic categories. The first group focuses on image quality assessment and diagnostic reliability in the context of AI, including domain shift analysis, motion compensation, and low-dose imaging strategies. The second group addresses image processing and reconstruction algorithms, examining how preprocessing, denoising, artifact correction, and geometric calibration influence image quality and diagnostic utility. The third group emphasizes clinical image quality and protocol optimization, evaluating whether the quality of specific acquisition strategies and reconstruction approaches is sufficient for reliable diagnosis in real-world clinical settings.

By integrating these perspectives, this Special Issue highlights that image quality assessment is not a peripheral technical exercise but a central component of evidence-based diagnostic imaging. The contributions collectively demonstrate that maintaining and objectively evaluating image quality are essential for ensuring diagnostic accuracy, patient safety, and clinical trust—particularly in an era defined by low-dose imaging, advanced reconstruction techniques, and rapidly expanding AI applications.

## 2. Conceptual and Clinical Framework of Image Quality Assessment

### 2.1. Medical Perspectives on Image Quality in Diagnostic Imaging and Need for Continued Research

Image quality is one of the most critical yet frequently underestimated determinants of diagnostic accuracy in modern medical imaging. From a clinical standpoint, image quality is not an abstract technical construct but a direct enabler of correct diagnosis, appropriate therapy planning, treatment monitoring, and, ultimately, patient outcomes. Image quality in medical imaging is a pivotal determinant of an image’s usefulness for detecting pathological alterations and assessing their extent. Image quality is tightly coupled to the quality of diagnosis, therapy planning and delivery, treatment monitoring, and rehabilitation efficacy. Accordingly, image quality can be defined as the degree to which an image enables the achievement of these clinical objectives.

### 2.2. Image Quality as a Clinical, Task-Driven Concept

Multiple factors contribute to image quality: selecting the appropriate imaging modality, properly defining the region of interest (ROI), ensuring the task-specific detectability of disease or injury, and aligning image properties with the demands of the clinical task (e.g., micro-calcification detection versus perfusion quantification). In everyday medical practice, diagnostic imaging is inherently task-specific. The requirements for detecting micro-calcifications in dental radiographs, identifying subtle periapical lesions, assessing perfusion deficits in CT, or evaluating tissue morphology in MRI fundamentally differ. Consequently, image quality cannot be reduced to a single universal metric. Instead, image quality reflects a balance among spatial resolution, contrast, noise, and artifact suppression, tailored to a particular diagnostic question [1,2,3,4].

### 2.3. Determinants of Image Quality Across Modalities

From a medical perspective, the core determinants of image quality—sharpness, contrast, noise, and artifacts—have tangible clinical consequences. Motion blur in CT thermography during ablation procedures can obscure treatment margins; low contrast-to-noise ratios in ultra-low-dose CT may hinder lesion detectability; geometric distortions in fluoroscopy can compromise the spatial measurements critical for functional assessment.

### 2.4. Technical Perspectives on Image Quality Assurance

From a technical perspective, a central concept is image clarity, i.e., how effectively clinically relevant information (anatomy, physiology, and function) is displayed. Image clarity is governed primarily by sharpness (the blurring of structural boundaries due to limited spatial resolution and/or motion). Sources of blurring include the detector or system point-spread function, sampling constraints (pixel size), and motion blur (patient or organ motion). Another factor affecting image quality is contrast (the signal difference between a target structure and its background). One distinguishes global versus local contrast; however, clinically, task-based contrast—the contrast between the pathology and its surroundings under specified acquisition and reconstruction conditions—is paramount. Image noise is the random signal fluctuations that degrade precision and conceal fine detail. Noise arises from quantum fluctuations (Poisson statistics in CT/PET), receiver/electronic noise (MRI/US), and reconstruction noise. Finaly, artifacts are systematic deviations from true anatomy/physiology that affect image quality. Examples of artifacts include CT beam-hardening (CT), partial-volume effects (MRI), ultrasound edge enhancement, and acoustic shadowing.

### 2.5. Beyond Classical Metrics: The Need for Advanced Quality Assessment

Image quality has been quantitatively assessed using classical metrics such as SNR and CNR, which are often suboptimal because they solely rely on ROI intensity and assume simple local noise statistics. Modern assessments therefore incorporate, e.g., task-based metrics that evaluate how well the system performs a specific clinical application, such as detecting micro-calcifications at a given dose, or texture-based measures that support objective quality evaluation [4].

### 2.6. Clinical Implications and Future Directions

From the clinician’s perspective, maintaining high image quality is essential for not only accurate diagnosis but also confidence in ruling out disease. High negative predictive value, as demonstrated in several clinical studies included in this Special Issue, directly affects patient triage, resource utilization, and clinical decision-making.

Machine and deep learning models are increasingly being applied in medical imaging. The publications in this group focus on the quality of imaging data and the impact of this quality on analytical outcomes, typically measured using metrics such as sensitivity, specificity, and AUC.

## 3. Three Groups of Publications in This Special Issue

A. Assessment of image quality and diagnostic reliability using AI

Kuschol [5] introduced DSMRI, a dedicated framework for analyzing domain shift across multi-center MRI datasets, a critical issue affecting the reliability and generalizability of medical imaging research. Domain shift arises from scanner variability, acquisition protocols, and site-specific differences, potentially compromising downstream analyses. DSMRI evaluates datasets by extracting a wide range of features from spatial, frequency, and wavelet domains, including texture-based metrics that capture structural and noise-related variation. The framework also employs t-SNE and UMAP visualizations to cluster similar data and highlight inter-site discrepancies. Quantitative measures—such as domain shift distance, classification accuracy, and feature rankings—provide objective insight into dataset heterogeneity. DSMRI was validated on seven large multi-site neuroimaging datasets, demonstrating a strong ability to quantify domain differences and support the development of harmonization and adaptation strategies. The tool addresses a key gap in MRI research by offering a systematic approach to identifying and characterizing domain shift.

Huflage [6] conducted a cadaveric study to evaluate the feasibility and image quality of ultra-low-dose unenhanced abdominal CT using photon-counting detector technology with tin filtration (Sn 100 kVp), which was compared with conventional 120 kVp imaging. Eight cadaver specimens were scanned at three radiation levels: standard (3 mGy), low (1 mGy), and ultra-low (0.5 mGy). Image quality was assessed through CNRs in renal cortex and subcutaneous fat, alongside subjective radiologist scoring. The results indicated that although the CNR decreased with radiation dose reduction, Sn 100 kVp consistently outperformed 120 kVp at all dose levels. Subjective scoring rated standard-dose images highest; at the ultra-low dose, tin-filtered imaging yielded higher perceived quality. Inter-reader reliability was high (ICC = 0.844). The study confirms that tin-filtered photon-counting CT enables meaningful dose reduction while preserving diagnostic value, offering promise for safer abdominal imaging.

Kostyrko [7] assessed multiple registration algorithms aimed at reducing motion artifacts in CT-thermography (CTT) during thermal ablation procedures. CTT involves repeated CT acquisitions at fixed intervals, hindering inter-scan motion. Using four porcine in vivo datasets obtained during microwave ablation, Kostyrko compared rigid, elastic, and combined registration techniques. Fifteen radiologists scored the resulting images. Statistical analysis (Friedman’s test) showed significant differences only in ablation-probe motion evaluation, where the accuracy of rigid registration was lower than that of elastic and combined approaches. Quantitative assessment confirmed significantly reduced probe-movement distance after combined registration. Elastic methods improved motion compensation, rigid registration better retained image stability in some contexts. Overall, the study highlights that combined registration strategies offer the most balanced solution, effectively minimizing motion artifacts and enhancing the interpretability of CTT during interventional procedures.

B. Image processing and reconstruction algorithms and methods

This group of publications primarily addresses methods for preprocessing images aimed at increasing overall image quality (e.g., low-dose CT reconstruction, denoising, super-resolution imaging, and artifact correction) as well as evaluating how these methods influence diagnostic utility. Such approaches considerably affect the quality of images across various modalities, e.g., in classifying median nerves in ultrasound images [8], estimating patient age and sex based on CT vertebral body scans [9], or characterizing aortic tissue in IVUS [10].

Ahn et al. [11] proposed AntiHalluciNet, an early-stage auditing tool designed to identify and quantify spurious structural artifacts—often called hallucinations—generated by deep learning (DL) denoising models in low-dose CT imaging. As DL denoising becomes increasingly common in medical imaging, concerns have arisen about unintended structural patterns that could mislead clinical interpretation. To address this issue, the authors constructed paired datasets of structure-embedded noise images and pure noise images, enabling AntiHalluciNet to learn distinctions between genuine and artificially introduced structures. A new metric, the Residual Structure Index (RSI), was proposed to measure AntiHalluciNet’s confidence in detecting structural components within residual noise. Using this index, the tool demonstrated the ability to not only identify hallucinated structures but also estimate image fidelity without requiring access to full reference images—an improvement over conventional metrics such as SSIM, which depend on paired datasets. The study highlights AntiHalluciNet’s potential as a practical auditing framework to monitor DL denoising behavior, enhance transparency, and reduce the risks of clinically harmful artifacts in low-dose CT workflows. Although preliminary, the results show that such tools could become integral to validating AI-based medical imaging systems and increasing reliability in safety-critical diagnostic settings.

Eda et al. [12] analyzed the three-dimensional morphometric characteristics of the volar lunate facet of the distal radius to further understand sex-based anatomical differences relevant to fracture fixation. Using 64 CT scans (34 women, 30 men), the authors reconstructed 3D bone models and measured multiple geometric parameters, including bone axis distance, volar–dorsal distance, radial–ulnar distance, 3D straight-line distance, inclination angle, and transverse diameter. The results showed that men have a larger transverse radius diameter and a more pronounced volar lunate facet protrusion than women, indicating a steeper volar surface inclination. This anatomical variation suggests that standard volar locking plates do not optimally fit male wrist anatomy, potentially requiring additional fixation strategies. The study highlights the importance of incorporating sex-specific morphology into preoperative planning and implant selection to improve fixation stability and surgical outcomes.

Cooper et al. [13] evaluated a self-calibrating bundle adjustment method to correct geometric distortions in single-plane fluoroscopy systems, which commonly experience distortions caused by hardware components and magnetic field interactions. The researchers conducted controlled experiments using three Philips Easy Diagnost R/F systems, capturing 18–22 images of a calibrated target field containing 121 pre-surveyed tantalum beads. By estimating five to eight distortion coefficients, the proposed method provided substantial improvements: 83–85% higher image point precision and 85–95% higher 3D reconstruction accuracy after calibration. The corrected images were then applied to analyze diaphragmatic motion and assess potential diagnostic use in detecting hemidiaphragm paralysis. The study demonstrates that self-calibrating distortion modeling can notably enhance the accuracy, reliability, and diagnostic value of fluoroscopic imaging, with possible applications in surgical navigation and broader clinical workflows.

C. Clinical image quality and the importance of imaging protocols

The last publication group discusses image quality in the context of clinical applications. The researchers examined whether a specific approach to performing imaging examinations such as X-ray, MRI, or ultrasound (including acquisition protocols, parameter selection, and evaluation methodology) provides sufficient image quality to enable reliable diagnosis or exclusion of pathology.

Lis et al. [14], in a retrospective pilot study, evaluated whether simple chest X-ray (CXR) measurements can be used to reliably screen for aortic arch dilation, using CT angiography (CTA) as the reference. The analysis included 240 emergency department patients who had undergone both CXR and CTA within seven days. Three aortic knob dimensions—horizontal, oblique, and vertical—were assessed, revealing that horizontal knob width most strongly correlated with CTA-derived aortic diameters at key anatomical levels. A regression model incorporating horizontal diameter, age, and sex achieved an AUC of 0.884, indicating strong discriminatory power. Using a conservative upper-bound threshold produced 100% sensitivity and 54% specificity, with a negative predictive value of 1.00, indicating the method is highly reliable for excluding dilation. The study suggests that integrating basic CXR measurements with demographic factors offer sa cost-effective, widely accessible screening tool, pending further validation across diverse populations.

Gohla et al. [15] compared two iterative reconstruction (IR) techniques—ADMIRE and SAFIRE—with traditional filtered back projection (FBP) in non-enhanced head CT at standard and simulated low-dose levels (90% and 70%). Based on the data from 21 patients, image noise spectra, Hounsfield Unit measurements, and qualitative radiologist scoring were analyzed. The image noise was significantly lower with both IR methods than with FBP (*p* < 0.001). ADMIRE performed best at low and high spatial frequencies, and SAFIRE was superior in the mid-frequency range. Radiologists consistently rated ADMIRE and SAFIRE higher than FBP in overall image quality, contrast, noise suppression, and detail across all dose levels. The findings confirm that IR techniques provide substantial qualitative and quantitative improvements, supporting dose reduction without compromising diagnostic reliability in routine neuroimaging.

Kim et al. [16] reviewed machine learning (ML) methods used for diagnosing human diseases, addressing their increasing relevance in medical decision-making. The authors highlight the growing diversity of ML algorithms capable of identifying complex patterns, predicting outcomes, and supporting clinicians across domains such as oncology, cardiology, neurology, and infectious diseases. The review emphasizes the advantages of ML—such as scalability, pattern discovery, and predictive accuracy—while noting challenges including interpretability, data heterogeneity, and the need for robust validation. The presented examples illustrate applications ranging from early disease detection to risk stratification. The authors compared algorithmic families, noting strengths and limitations of methods such as decision trees, neural networks, support vector machines, and ensemble models. Overall, the review provides a comprehensive synthesis guiding researchers and clinicians in selecting the appropriate machine learning approaches for specific medical contexts.

Malik et al. 2024 [17] reviewed CT diagnostic reference levels (DRLs) in India in comparison with international standards. DRLs help optimize radiation exposure; yet, the study reveals wide variability internationally (differences up to 50%) and within India among states. For head CT, Indian DRLs were 20–30% lower (27–47 mGy) than many global benchmarks (~60 mGy). For abdominal CT, however, Indian DRLs were 10–15% higher (12–16 mGy) than reported international averages (~13 mGy). The contributors to variability include imaging protocols, equipment age, patient characteristics, and regulatory discrepancies. The review highlights that effective dose optimization strategies—such as automatic exposure control and iterative reconstruction—are capable of reducing radiation by 25–60% without compromising image quality. Lessons from international programs, such as the UK’s historically successful DRL-driven 30% dose reduction, indicate that India could achieve 20–40% dose reductions through systematic standardization and best practice adoption.

## 4. Conclusions

Image quality is not merely a technical attribute of diagnostic images but a central clinical determinant of diagnostic reliability and patient care. The research presented in this Special Issue highlights that sustained, methodologically rigorous investigation into image quality assessment is indispensable—particularly in an era defined by low-dose imaging, advanced reconstruction, and artificial intelligence. Maintaining and increasing image quality are therefore medical responsibilities and scientific priorities.

## Data Availability

Not applicable.

## Abbreviations

The following abbreviations are used in this manuscript:

AI	Artificial Intelligence
AUC	Area Under the Curve
CNR	Contrast-to-Noise Ratio
CT	Computed Tomography
CTA	Computed Tomography Angiography
CTT	Computed Tomography Thermography
CXR	Chest X-Ray
DL	Deep Learning
DRL	Diagnostic Reference Level
FBP	Filtered Back Projection
ICC	Intraclass Correlation Coefficient
IR	Iterative Reconstruction
LD	Low Dose (
MRI	Magnetic Resonance Imaging
PET	Positron Emission Tomography
ROI	Region of Interest
RSI	Residual Structure Index
SAFIRE	Sinogram-Affirmed Iterative Reconstruction
SNR	Signal-to-Noise Ratio
SSIM	Structural Similarity Index Measure
t-SNE	t-Distributed Stochastic Neighbor Embedding
UMAP	Uniform Manifold Approximation and Projection
US	Ultrasound
